# Australian Research Related to Sporting and Musculoskeletal Injuries of the Foot and Ankle: A Bibliometric Analysis

**DOI:** 10.1002/jfa2.70184

**Published:** 2026-07-08

**Authors:** Benjamin Peterson, John Osborne, Ameer Nor Azhar, Matthew Carroll, Peta Tehan

**Affiliations:** ^1^ School of Health Medical and Applied Sciences CQUniversity Rockhampton Queensland Australia; ^2^ Central Queensland University S.P.O.R.T. Research Cluster CQUniversity Rockhampton Queensland Australia; ^3^ School of Primary and Allied Health Care Monash University Frankston Victoria Australia; ^4^ Latrobe University Bundoora Victoria Australia; ^5^ Auckland University of Technology Auckland New Zealand; ^6^ School of Clinical Sciences at Monash Health Monash University Clayton Victoria Australia

**Keywords:** ankle, biomechanics, foot, musculoskeletal, publications, research, sports

## Abstract

**Background:**

There is growing research evidence related to musculoskeletal and sports podiatry published by Australian researchers. This short report presents data from the musculoskeletal and sports podiatry stream of a national bibliometric review which aimed to map all Australian podiatry‐related research from 1970 to 2024.

**Methods:**

A systematic search of the literature was conducted via Scopus until December 2024. Studies were screened for eligibility using Covidence. Study meta‐data was analysed using Biblioshiny to describe publications volume, authors, institutions, journals, and research collaborations. Via manual processes, each publication was categorised for: level of evidence using National Health and Medical Research Council criteria; research type using the United Kingdom Clinical Research Collaboration Health Research Classification System; and funding source, using Higher Education Research Data Collection specifications.

**Results:**

The search strategy yielded 288 records published by 774 authors (19% international), with a total 12,593 citations, with 44.1 mean citations per article from 1991 to 2024 were included. A total of 65 articles (23%) were categorised as level I evidence. The British Journal of Sports Medicine and the Journal of Science and Medicine in Sport were equally the most frequent publication sources, publishing 23 (8%) articles respectively. Podiatry‐related musculoskeletal and sports research is usually undertaken without dedicated funding (67%). The majority of published articles focussed on the evaluation of treatments and therapeutic interventions (36%) and on the aetiology of conditions (34%).

**Conclusion:**

The musculoskeletal and sports podiatry stream was the most widely researched, with the highest proportion of level I evidence among all streams of the national bibliometric review. Given the high burden of disease and adverse impacts on activities of daily living associated with musculoskeletal conditions, further attention in preventative care and the promotion of wellbeing in sports and musculoskeletal research would be beneficial.

## 1 Background

Lower limb musculoskeletal and sporting conditions, and lower limb biomechanical function, are important facets of podiatric practice. Musculoskeletal pain of the foot and ankle has a reported prevalence of 17% in the general population [1]. The prevalence of injury is higher among sporting populations, for example, the prevalence of foot and ankle injuries in runners ranges between 7.8%–35% and 6.1%–34.2% respectively [2]. Pain and injury of the foot and ankle affects participation in recreational and physical activities [3], and is associated with an increased risk of falls [4], negative psychological constructs [5], and reduced quality of life [6].

Management of musculoskeletal injuries is an important component of podiatry practice [7]. Podiatrists are trained to assess, diagnose, and manage musculoskeletal injuries of the foot, ankle, and lower limb [7]. An increased risk of specific injuries more proximal to the foot and ankle may also be attributed to atypical foot and lower limb biomechanical function [8, 9], although evidence is conflicting [10, 11]. Meanwhile, podiatry‐delivered interventions, such as the prescription of exercise [12], foot orthoses [13, 14], specific footwear [15], and prescription of scheduled medicines (such as corticosteroids, for podiatrists with endorsement for scheduled medicines) [16] may be used with the aim to improve pain and/or functional outcomes for individuals living with foot and ankle‐related musculoskeletal conditions, pain, or injuries.

There is a growing body of evidence, produced by researchers at Australian institutions, on musculoskeletal and sporting conditions of the foot and lower limb, advancing knowledge of the aetiology, diagnosis, prevention, and management of high‐burden lower‐limb problems. This evidence base has not yet been synthesised. This short report is part of a national research programme that aims to map current podiatry‐relevant research streams [17]. This will inform an Australian Delphi process to set national research priorities in podiatry, which is essential to improve outcomes in people who have musculoskeletal and sports‐related conditions. Therefore, this study reports the findings of the musculoskeletal and sports podiatry stream of a comprehensive bibliometric analysis of Australian podiatry research. The objective of this bibliometric analysis was to identify the levels and types of research being undertaken in podiatry‐related sports and musculoskeletal research, the authors undertaking this work and their affiliations, and the sources of research funding supporting this area of research in Australia.

## 2 Method

This study was a bibliometric analysis of articles published from January 1970 to December 2024 and indexed in Scopus. The Scopus database was selected due to its broader coverage of journals when compared with PubMed and Web of Science [18, 19], and its congruence with Biblioshiny application (version 5) (R version 4.4.3, Bibliometrix package; University of Naples Federico II, Naples, Italy, 2016). Scopus also offers the ability to search by publication, author or affiliation.

### 2.1 Search Strategy and Study Selection

The electronic search strategy applied to Scopus (Supporting Information S1) included three concepts: (i) lower limb pain, injury, function, or deformity, (ii) lower limb anatomical structures, (iii) sport and gait.

Results of the Scopus search were downloaded and imported into the online systematic review management platform Covidence (Veritas Health Innovation, Melbourne, Australia). Following removal of duplicates, five researchers (BP, JO, AN, MC, and PT) independently screened titles, abstracts, and full‐text articles of all retrieved articles, with each title screened by two researchers. Where required, conflicts were resolved via discussion or were arbitrated by PT or MC. Inclusion criteria were considered: original articles or systematic reviews published in English, completed at an Australian education or healthcare institution, in an Australian cohort of participants, where at least one author had an Australian affiliation. For systematic reviews, the first or last author was required to have had an Australian affiliation. Biomechanical laboratory‐based studies that investigated foundational principles and functional relationships were excluded due to their limited direct applicability to clinical practice. Similarly, lab‐based investigations in controlled environments, using specialised instrumentation, or studies that captured kinematics or kinetics, plantar pressures or electromyography were also excluded. Given the breadth of musculoskeletal and sports research relevant to podiatry, the eligibility criteria were narrowed to include only foot and ankle research and studies conducted predominantly in adult populations. A separate paediatric analysis has been published separately, which includes paediatric musculoskeletal conditions [20]. Studies were excluded if they were: guidelines, consensus documents, case studies, research letters, editorials, commentaries, or conference abstracts.

### 2.2 Data Extraction

Meta‐data from all included studies were extracted using Biblioshiny [21]. Extracted bibliometric measures were: publication year, journal title, number of citations (as reported in the Scopus database (Elsevier, Amsterdam, Netherlands)), author details, number of study authors, and authors' institutional and country affiliations.

### 2.3 Data Synthesis

The level of evidence, according to the National Health and Medical Research Council (NHRMC) criteria, of each included study was manually assigned by a single reviewer (either BP or JO). NHMRC guidelines for grading of levels of evidence include six levels, and study designs designated for classification at each level vary according to the type of research question. Similarly, research focus was categorised according to the United Kingdom Clinical Research Commission (UKCRC) Health Research Classification System [22]. The UKCRC system classifies research activity types against 48 codes within eight broad categories: (i) underpinning research, (ii) aetiology, (iii) prevention of disease and conditions, and promotion of well‐being, (iv) detection, screening, and diagnosis, (v) development of treatments and therapeutic interventions, (vi) evaluation of treatments and therapeutic interventions, (vii) management of diseases and conditions, (viii) health and social care services research. Funding categories were extracted from each study, where reported, following the Australian Government Higher Education Research Data Collection (HERDC) specifications as follows: (i) category 1: Australian competitive grant research and development income; (ii) category 2: other public sector research and development income; (iii) category 3: industry and other research and development income; (iv) category 4: cooperative research centre research and development income [23].

## 3 Results

### 3.1 Article Characteristics

**FIGURE 1:**
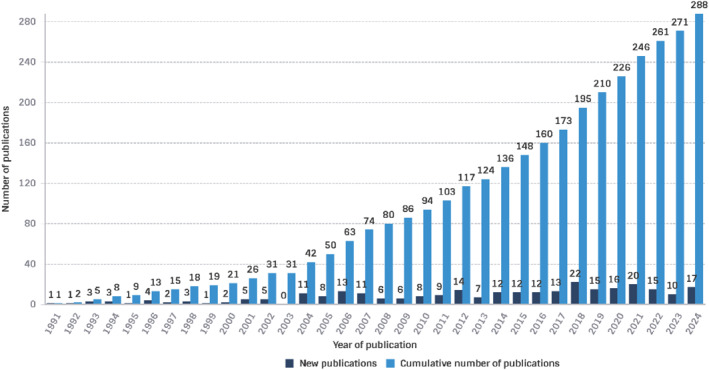
Number of articles (per year and cumulative).

The search strategy returned 3087 original records. Full‐text records were accessed for 757 articles, of which 288 were included in this review (Supporting Information S2). Table 1 presents the characteristics of included studies. The British Journal of Sports Medicine and Journal of Science and Medicine in Sport had the highest volume of published literature, both with 23 articles each (8%) (Table 2). The peak year for research output was 2018, with 22 eligible studies published. Figure 1 illustrates the number of sports and musculoskeletal podiatry research published from 1991 to 2024 (cumulative and per year).

**TABLE 1:** Publication characteristics.

Years	1991–2024
Total number of articles	288
Original articles	233
Systematic reviews	55
Mean years from publication	12.6
Mean citations per article	44.1
Citations	12,703
Total authors	774
Mean co‐authors per article	4.62
International co‐authorships (%)	19.44
Single‐authored publications	7

**TABLE 2:** Top five most frequent journals.

Journal		*n* (%)	Impact factor 2024
1.	*British Journal of Sports Medicine*	23 (8)	16.2
1.	*Journal of Science and Medicine in Sport*	23 (8)	3.4
2.	*Journal of Foot and Ankle Research*	21 (7)	2.2
3.	*Gait and Posture*	14 (5)	2.4
4.	*BMC Musculoskeletal Disorders*	12 (4)	2.4
4.	*Journal of Orthopaedic and Sports Physical Therapy*	12 (4)	5.8
5.	*Journal of the American Podiatric Medical Association*	11 (4.2)	0.6
5.	*Physical Therapy in Sport*	11 (3.6)	2.4

### 3.2 Authors, Institutions and Countries

The included studies were published by 774 authors (4.62 co‐authors per article) (Table 1). The top 10 most represented authors are outlined in table three, with Menz HB (*n* = 46), Landorf KB (*n* = 30), and Hiller CE (*n* = 23) yielding the highest output (Table 3). The top five most represented institutions are presented in Table 4, being La Trobe University, University of Sydney, University of Queensland, University of Newcastle, Monash University and University of Melbourne.

**TABLE 3:** Top 10 most frequent authors.

Author		*n* (%)
1.	Menz HB	46 (16)
2.	Landorf KB	30 (10)
3.	Hiller CE	23 (8)
4.	Vicenzino B	22 (8)
4.	Refshauge KM	22 (8)
5.	Munteanu SE	19 (7)
6.	Burns J	15 (5)
7.	Adams R	11 (4)
7.	Whittaker GA	11 (4)
8.	Steele JR	10 (3)
8	Kilbreath SL	10 (3)
9	Bonanno DR	9 (3)
10	Herbert RD	8 (3)

**TABLE 4:** Top five most frequent institutional affiliations.

Institution		*n*
1.	La Trobe University	116
2.	University of Sydney	87
3.	University of Queensland	35
4	University of Newcastle	26
4.	Monash University	26
5.	University of Melbourne	19

### 3.3 Citations

The top 10 most highly cited articles according to total citations and number of citations per year are presented in Table 5. The articles with the highest number of total citations were (i) an observational study of rates and risk factors for ankle injuries in basketball [24], (ii) a validity and reliability study of the Cumberland Ankle Instability tool [25], and (iii) a prospective cohort study of the incidence and distribution of stress fractures among competitive track and field athletes [26].

**TABLE 5:** Top 10 cited articles, expressed as total citations and citations/year.

Article		Total citations	Article		Citations/year
1.	McKay, G. D., Goldie, P. A., Payne, W. R., & Oakes, B. W. (2001). Ankle injuries in basketball: Injury rate and risk factors. *British journal of sports medicine*, *35*(2), 103–108.	500	1.	Clemson, L., Fiatarone Singh, M. A., Bundy, A., Cumming, R. G., Manollaras, K., O'Loughlin, P., et al. Integration of balance and strength training into daily life activity to reduce rate of falls in older people (the LiFE study): Randomised parallel trial. *British medical journal*. 2012; 345:e4547.	21.30
2.	Hiller, C. E., Refshauge, K. M., Bundy, A. C., Herbert, R. D., & Kilbreath, S. L. (2006). The Cumberland ankle instability tool: a report of validity and reliability testing. *Archives of physical medicine and rehabilitation*, *87*(9), 1235–1241.	427	2.	Hiller, C. E., Refshauge, K. M., Bundy, A. C., Herbert, R. D., & Kilbreath, S. L. (2006). The Cumberland ankle instability tool: a report of validity and reliability testing. *Archives of physical medicine and rehabilitation*, *87*(9), 1235–1241.	20.30
3.	Bennell, K. L., Malcolm, S. A., Thomas, S. A., Wark, J. D., & Brukner, P. D. (1996). The incidence and distribution of stress fractures in competitive track and field athletes. A twelve‐month prospective study. *The American journal of sports medicine*, *24*(2), 211–217.	340	3.	McKay, G. D., Goldie, P. A., Payne, W. R., & Oakes, B. W. (2001). Ankle injuries in basketball: Injury rate and risk factors. *British journal of sports medicine*, *35*(2), 103–108.	19.20
4.	Clemson, L., Fiatarone Singh, M. A., Bundy, A., Cumming, R. G., Manollaras, K., O'Loughlin, P., et al. Integration of balance and strength training into daily life activity to reduce rate of falls in older people (the LiFE study): Randomised parallel trial. *British medical journal.* 2012; 345:e4547	319	4.	Hiller, C. E., Kilbreath, S. L., & Refshauge, K. M. (2011). Chronic ankle instability: Evolution of the model. *Journal of athletic training*, *46*(2), 133–141.	15.00
5.	Yates, B., & White, S. (2004). The incidence and risk factors in the development of medial tibial stress syndrome among naval recruits. *The American journal of sports medicine*, *32*(3), 772–780.	256	5.	Crossley KM, Patterson BE, Culvenor AG*,* et al. Making football safer for women: a systematic review and meta‐analysis of injury prevention programmes in 11 773 female football (soccer) players. *British journal of sports medicine* 2020;54:1089–1098.	12.30
6.	Buchbinder, R., Ptasznik, R., Gordon, J., Buchanan, J., Prabaharan, V., & Forbes, A. (2002). Ultrasound‐guided extracorporeal shock wave therapy for plantar fasciitis: A randomized controlled trial. *Journal of American medical association*, *288*(11), 1364–1372.	255	6.	Thompson, C., Schabrun, S., Romero, R., Bialocerkowski, A., van Dieen, J., & Marshall, P. (2018). Factors Contributing to Chronic Ankle Instability: A Systematic Review and Meta‐Analysis of Systematic Reviews. *Sports medicine*, *48*(1), 189–205.	11.90
7.	Hiller, C. E., Kilbreath, S. L., & Refshauge, K. M. (2011). Chronic ankle instability: Evolution of the model. *Journal of athletic training*, *46*(2), 133–141.	240	7.	Yates, B., & White, S. (2004). The incidence and risk factors in the development of medial tibial stress syndrome among naval recruits. *The American journal of sports medicine*, 32(3), 772–780.	11.10
8.	Bennett, P. J., Patterson, C., Wearing, S., & Baglioni, T. (1998). Development and validation of a questionnaire designed to measure foot‐health status. *Journal of the American podiatric medical association*, *88*(9), 419–428.	234	8.	Irving, D. B., Cook, J. L., & Menz, H. B. (2006). Factors associated with chronic plantar heel pain: a systematic review. *Journal of science and medicine in sport*, 9(1–2), 11–24.	12.13
8	Irving, D. B., Cook, J. L., & Menz, H. B. (2006). Factors associated with chronic plantar heel pain: a systematic review. *Journal of science and medicine in sport*, *9*(1–2), 11–24.	234	9.	Bennell, K. L., Malcolm, S. A., Thomas, S. A., Wark, J. D., & Brukner, P. D. (1996). The incidence and distribution of stress fractures in competitive track and field athletes. A twelve‐month prospective study. *The American journal of sports medicine*, 24(2), 211–217.	11.00
9=	Vicenzino B.; Branjerdporn M.; Teys P.; Jordan K. (2006). Initial changes in posterior talar glide and dorsiflexion of the ankle after mobilization with movement in individuals with recurrent ankle sprain. *Journal of orthopaedic and sports physical therapy*, 36 (7) 464–471.	175	10	Buldt, A. K., Allan, J. J., Landorf, K. B., & Menz, H. B. (2018). The relationship between foot posture and plantar pressure during walking in adults: A systematic review. *Gait & posture*, 62, 56–67.	11.00
9=	Malliaras P.; Cook J.L.; Kent P. (2006). Reduced ankle dorsiflexion range may increase the risk of patellar tendon injury among volleyball players. *Journal of science and medicine in sport.* (9) 304–309	175			
10	Green T.; Refshauge K.; Crosbie J.; Adams R. A randomized controlled trial of a passive accessory joint mobilization on acute ankle inversion sprains. *Physical therapy*, (81) 984–994	160			

### 3.4 Research Types and Level of Evidence

According to the UKCRC criteria, 103 (36%) articles were focussed on evaluation of treatments and therapeutic interventions, 103 (36%) on aetiology, 54 (19%) on detection, screening, and diagnosis, and 23 (8%) on management of diseases and conditions. The remaining articles focussed on health and social care services research (*n* = 5, 2%), development of treatments and therapeutic interventions (< 1%), underpinning research (< 1%), and prevention of disease and conditions and promotion of well‐being (< 1%). According to the NHMRC levels of evidence, 65 of 308 articles (23%) provided level I evidence, 69 (24%) provided level II evidence, 90 (31%) provided level III evidence, and 65 (23%) provided level IV evidence.

### 3.5 Sources of Funding

Most included studies reported no research funding (*n* = 195, 68%). Of the funded studies (*n* = 91, 32%), 40 (14%) were supported by category 1 funding, 23 (8%) by category 2 funding, and 28 (10%) by category 3 funding. No study reported receipt of category 4 funding.

## 4 Discussion

This study was a bibliometric analysis of sports and musculoskeletal research related to podiatry. In this study, the authors synthesised the types of research outputs, levels of evidence, sources of research funding, citation metrics, and authorship details of eligible studies published from 1991 to 2024. This synthesis identified that sports and musculoskeletal podiatry is the most widely researched facet of podiatry practice [17]. While there were a number of highly prolific researchers represented in the authorship lists of eligible studies, the data indicate that research in this speciality is driven by the collective efforts of many authors across a wide range of institutions, usually without dedicated funding.

The total number of musculoskeletal and sports‐related research papers was the highest among all fields represented in our larger bibliometric analysis. This points to a growing research interest in this area of lower‐limb health across a range of professions. Musculoskeletal conditions are the leading cause of disability and healthcare financial burden in Australia and worldwide [27], with an estimated 7.3 million Australians living with musculoskeletal conditions [28]. Therefore, there have been calls to prioritise musculoskeletal research [29]. There has been growth in research output over time, and the five highest years of research output have all occurred since 2018. Within the period of 2018–2024, there were years of reduced productivity (particularly 2023), which have not been explained by the findings of this bibliometric analysis, but speculatively may have been related to lockdown measures, lab closures, and pauses in face‐to‐face data collection during 2020 and 2021 as a consequence of COVID‐19 [30].

Podiatry‐related musculoskeletal and sports research was published across a wide range of journals, with most studies not published in foot and ankle‐focused journals. Studies published in the traditional podiatry literature were cited less frequently. In fact, only one of the top 10 most cited papers (all time) and none of the most cited papers by year were published in podiatry‐specific journals. This finding points to the need for podiatrists to consult a range of journals when reviewing contemporary research in musculoskeletal and sports podiatry. Meanwhile, this may further suggest a need for researchers and professional organisations to promote new research findings to clinicians and may support an argument to strive for open access publishing, where institutional and funding constraints allow. Ensuring that research findings are accessible to podiatrists is important for translating published research into real‐world patient outcomes.

The findings of this bibliometric analysis should be interpreted in the context of certain limitations. First, the classification of musculoskeletal and sports research was constrained by the absence of a universally accepted definition; as a result, we applied a pragmatic definition encompassing clinically relevant research on the adult foot and ankle in musculoskeletal and sports research. Furthermore, limiting eligibility to only studies of the foot and ankle, means that the findings of this synthesis has excluded a range of studies with direct relevance to podiatry practice in Australia, such as those related to assessment, function, or pathology of the knee or the hip. Exclusion of pre‐clinical and laboratory‐based studies means that many relevant studies related to lower limb biomechanics have not been represented within this synthesis. These limitations to the eligibility criteria may have influenced the relative proportion of research types and/or levels of evidence. Second, there are challenges in assigning articles to a single research category, particularly given the interdisciplinary nature of many studies. Third, as coding of research categorisation, levels of evidence, and funding sources was a manual process completed by multiple authors, there is the potential that some studies may have been incorrectly coded or misclassified. Measures were taken to mitigate this risk, including checking and cleaning of data by PT and MC. Fourth, as citation metrics are influenced by citation age, these may not fully represent the ongoing or future impact of included studies. Finally, synthesis of sources of research funding was reliant on authors funding declarations, therefore may not accurately represent the actual financial investment (project support or investigator salaries) of research in this stream.

## 5 Conclusion

Musculoskeletal and sports podiatry is the most widely researched stream of Australian podiatry research. This stream of podiatry research is usually undertaken without dedicated funding, and the majority of published articles focus on the evaluation of interventions and on the aetiology of conditions. Musculoskeletal and sports podiatry research output in Australia is driven by many academics, and one in five studies has been conducted in collaboration with international collaborators. Future bibliometric analyses of this research stream should utilise broader eligibility criteria to more accurately reflect the breadth of musculoskeletal and sports podiatry‐related research conducted by researchers within Australian institutions.

## Author Contributions

**Benjamin Peterson:** data curation, formal analysis, writing – original draft. **John Osborne:** data curation, formal analysis, writing – review and editing. **Ameer Nor Azhar:** data curation, formal analysis, writing – review and editing. **Matthew Carroll:** formal analysis, software, writing – review and editing. **Peta Tehan:** conceptualization, data curation, formal analysis, writing – review and editing.

## Funding

This work was supported by Australian Podiatry Education and Research Foundation.

## Ethics Statement

The authors have nothing to report.

## Consent

The authors have nothing to report.

## Conflicts of Interest

The authors declare no conflicts of interest.

## Supporting information


Supporting Information S1

Supporting Information S2

## Data Availability

The data that supports the findings of this study are available in the supplementary material of this article.
