# Supplementary material for: Australian Research Related to Sporting and Musculoskeletal Injuries of the Foot and Ankle: A Bibliometric Analysis
Source: J Foot Ankle Res. 2026 Jul 8;19(3):e70184. doi: 10.1002/jfa2.70184 (PMC13345985; doi:10.1002/jfa2.70184)
Supplement: Supplementary file 2 — Supporting Information S2 [file JFA2-19-e70184-s001.docx]

**Sports and MSK Search Strategy**

| **Sports and MSK** |
| --- |
| String/concept 1: ((Foot OR ankle OR hip OR knee) w/5 (deformity OR pain OR injury)) OR ((Foot OR ankle OR hip OR knee) w/5 (tendon OR ligament OR bone OR muscle)) OR Sport* OR gait OR running  String/concept 2: Rehab* OR therap* OR prevent* OR footwear OR shoe* OR insole* OR ortho* OR biomech* OR assess* OR function OR performance  String/concept 3: Foot OR feet OR hip OR knee OR ankle OR pod*  Concept: 1 AND 2 AND 3 |
